# Birth prevalence of phenylalanine hydroxylase deficiency: a systematic literature review and meta-analysis

**DOI:** 10.1186/s13023-021-01874-6

**Published:** 2021-06-03

**Authors:** Pamela K. Foreman, Andrea V. Margulis, Kimberly Alexander, Renee Shediac, Brian Calingaert, Abenah Harding, Manel Pladevall-Vila, Sarah Landis

**Affiliations:** 1 BioMarin Pharmaceutical Inc, 770 Lindaro Street, San Rafael, CA 94901 USA; 2 RTI Health Solutions, Barcelona, Av. Diagonal 605, 9-4, 08028 Barcelona, Spain; 3 RTI Health Solutions, North Carolina, 3040 East Cornwallis Road, P.O. Box 12194, Research Triangle Park, NC 27709-2194 USA; 4 BioMarin (U.K.) Limited, 10 Bloomsbury Way, London, WC1A 2SL UK

**Keywords:** Phenylketonuria, Hyperphenylalaninemia, Prevalence, Newborn screening, Phenylalanine hydroxylase deficiency

## Abstract

**Background:**

Phenylalanine hydroxylase (PAH) deficiency is an autosomal recessive disorder that results in elevated concentrations of phenylalanine (Phe) in the blood. If left untreated, the accumulation of Phe can result in profound neurocognitive disability. The objective of this systematic literature review and meta-analysis was to estimate the global birth prevalence of PAH deficiency from newborn screening studies and to estimate regional differences, overall and for various clinically relevant Phe cutoff values used in confirmatory testing.

**Methods:**

The protocol for this literature review was registered with PROSPERO (International prospective register of systematic reviews). Pubmed and Embase database searches were used to identify studies that reported the birth prevalence of PAH deficiency. Only studies including numeric birth prevalence reports of confirmed PAH deficiency were included.

**Results:**

From the 85 publications included in the review, 238 birth prevalence estimates were extracted. After excluding prevalence estimates that did not meet quality assessment criteria or because of temporal and regional overlap, estimates from 45 publications were included in the meta-analysis. The global birth prevalence of PAH deficiency, estimated by weighting regional birth prevalences relative to their share of the population of all regions included in the study, was 0.64 (95% confidence interval [CI] 0.53–0.75) per 10,000 births and ranged from 0.03 (95% CI 0.02–0.05) per 10,000 births in Southeast Asia to 1.18 (95% CI 0.64–1.87) per 10,000 births in the Middle East/North Africa. Regionally weighted global birth prevalences per 10,000 births by confirmatory test Phe cutoff values were 0.96 (95% CI 0.50–1.42) for the Phe cutoff value of 360 ± 100 µmol/L; 0.50 (95% CI 0.37–0.64) for the Phe cutoff value of 600 ± 100 µmol/L; and 0.30 (95% CI 0.20–0.40) for the Phe cutoff value of 1200 ± 200 µmol/L.

**Conclusions:**

Substantial regional variation in the birth prevalence of PAH deficiency was observed in this systematic literature review and meta-analysis of published evidence from newborn screening. The precision of the prevalence estimates is limited by relatively small sample sizes, despite widespread and longstanding newborn screening in much of the world.

**Supplementary Information:**

The online version contains supplementary material available at 10.1186/s13023-021-01874-6.

## Background

Phenylalanine hydroxylase (PAH) deficiency is an autosomal recessive disorder that results in elevated concentrations of the amino acid phenylalanine (Phe) in the blood [1–4]. Over 1000 PAH variants exist [5], and depending on the inherited alleles, affected individuals may have very mild to pronounced elevation of Phe [4]. Phenylalanine hydroxylase catalyzes the conversion of Phe into tyrosine and is key to maintaining a stable concentration of Phe in the blood [7]. When PAH activity is decreased, blood Phe concentration increases from the typical mean of 60 μmol/L [3]. In addition, an estimated 1–2% of cases of hyperphenylalanemia (HPA) are secondary to a deficiency in tetrahydrobiopterin (BH4), a necessary cofactor for PAH and other amino acid-metabolizing enzymes [4, 6]. Cases of mutations in a heat shock co-chaperone family member, DNAJC12 have been also reported to result in HPA [8]. If left untreated, the accumulation of Phe can result in profound neurocognitive disability [2]. Early diagnosis and intervention are essential to preserve cognitive function [1, 3].

Treatment guidelines recommend initiation of treatment as early as possible upon diagnosis of PAH deficiency [3]. Treatment options include dietary and pharmaceutical management. Dietary management involves severely restricted intake of Phe (and protein)-rich foods based on each individual’s maximum Phe tolerance [9, 10] in combination with medical foods to supplement inadequate intake of protein and other essential nutrients due to the Phe-restricted diet. Approved pharmaceutical treatments for PAH deficiency include pegvaliase and sapropterin. While pegvaliase, a Phe-metabolizing enzyme composed of pegylated recombinant phenylalanine ammonia lyase, is approved for use only in adults (United States) and persons aged 16 years and above (Europe) who have uncontrolled Phe in blood (> 600 uM/L) with current treatment [11, 12], sapropterin dihydrochloride, a synthetic form of BH4, is indicated for use in children (> 1 month of age) and adults with BH4-responsive PKU in conjunction with a Phe-restricted diet [2, 13, 14].

Phenylalanine hydroxylase deficiency is classified into mild HPA, mild phenylketonuria (PKU), moderate PKU, and classical PKU based on blood Phe concentration obtained in the neonatal period (Table 1); however, concentrations determined in this period are unlikely to reflect peak untreated levels, as neonates vary in their dietary exposure to Phe before the blood sample is taken, and early treatment often precludes obtaining more definitive Phe concentrations [1].

**Table 1:** Current classification and treatment guidelines for PAH deficiency

Classification	Pretreatment blood phenylalanine concentration	Treatment recommended?	
		European guidelines^a^	ACMG^b^
Classical PKU	> 1200 µmol/L (> 20 mg/dL)	Yes	Yes
Moderate PKU	900–1200 µmol/L (15–20 mg/dL)	Yes	Yes
Mild PKU	600–900 µmol/L (10–15 mg/dL)	Yes	Yes
Mild HPA-gray zone	360–600 µmol/L (6–10 mg/dL)	Yes (only if < 12 years or in women before/during pregnancy)	Yes^c^
PAH deficiency not requiring treatment	120–360 µmol/L (2–6 mg/dL)	No	No

ACMG = American College of Medical Genetics and Genomics; HPA = hyperphenylalaninemia; PAH = phenylalanine hydroxylase; PKU = phenylketonuria

^a^van Wegberg et al. [2]

^b^Vockley et al. [3]

^c^After reviewing controversy regarding mixed treatment results with parents

Because of the severe consequences of untreated phenylalanine hydroxylase deficiency, many countries currently perform routine newborn screening for elevated blood Phe concentration [15–17]. Methods for measuring Phe have evolved over time, with increasing accuracy, initiating with the bacterial inhibition assay (Guthrie test) in 1963 [18] to the current state-of-the-art tandem mass spectrometry [19]. The Guthrie test has been suggested to miss as many as 1 in 25 affected newborns screened at or before 3 days of age [20].

The accumulation of data from newborn screening programs with varied screening methods employed across the world provides an opportunity to evaluate the birth prevalence of HPA and PKU at the regional and global levels. Here, we systematically review the published literature and analyze regional differences in HPA and PKU birth prevalence, overall and for various clinically relevant blood Phe concentration cutoff values used in confirmatory testing.

## Methods

The protocol for this literature review was registered with PROSPERO (International prospective register of systematic reviews: https://www.crd.york.ac.uk/PROSPERO/display_record.php?RecordID=156377; ID 156377).

### Birth prevalence

For the purpose of this review and to ensure consistent methodology in calculation of birth prevalence estimates across studies, birth prevalence was defined as cases identified during newborn screening divided by the number of newborns screened. This method was most frequently described in studies reporting birth prevalence of PAH deficiency from newborn screening programs.

### Literature search

PubMed and Embase were searched using a strategy based on the PICOS (population, intervention, comparison, outcomes, study design) framework (Additional file 1: Table A-1) [21]. The search strategy included terms to identify newborns, prevalence, incidence, newborn screening, Guthrie and other tests, PKU, HPA, and PAH deficiency (Additional file 1: Table A-2 and Table A-3). No language or time limits were implemented. Animal studies, editorials, and commentaries were excluded.

### Study selection

**Fig. 1:**
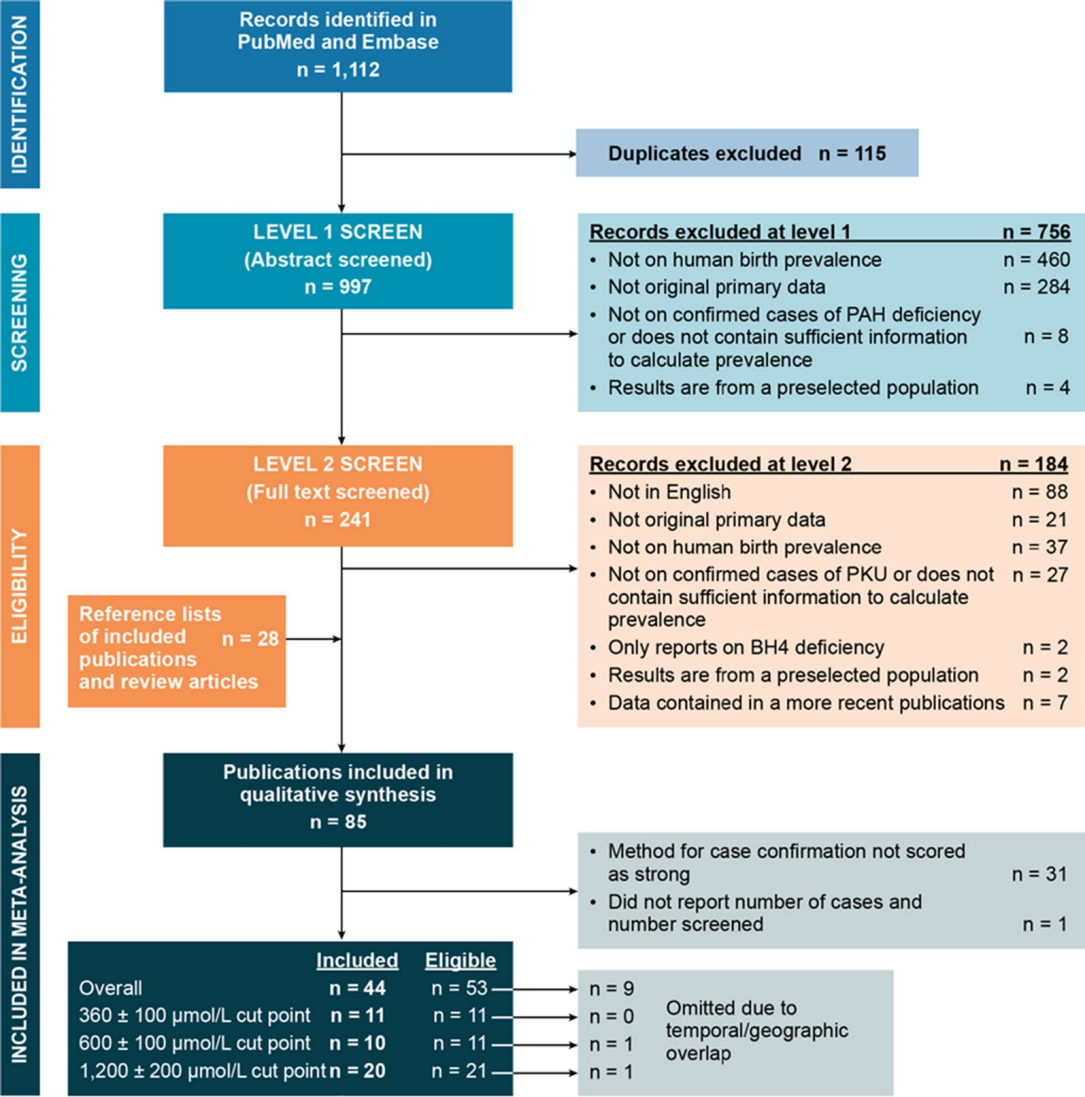
Study selection process. PRISMA chart modeled after Moher et al. [21]. BH4 = tetrahydrobiopterin; PAH = phenylalanine hydroxylase deficiency; PKU = phenylketonuria

Entries retrieved from PubMed and Embase were screened in two steps (Fig. 1): in level 1 screening, two researchers independently reviewed titles and abstracts; in level 2 screening, two researchers independently reviewed full-text articles. Lack of agreement on inclusion was resolved by discussion and consensus within the research team.

In level 1 screening (Additional file 1: Table A-4), conference abstracts, studies reporting exclusively on BH4 deficiency but not on PAH deficiency, and studies that focused primarily on assay development and/or validation were excluded. Publications were eligible if the abstract or title indicated that the paper presented original research and contained numeric reports on the birth prevalence of PAH deficiency. Birth prevalence must have been reported on an unselected population (e.g., studies on institutionalized patients were not eligible) and was required to be directly measured (rather than estimated from models). When duplicate records reporting on one study were identified, only one was retained; in this circumstance, records published in English were preferred.

The following additional criteria were applied in level 2 screening (Additional file 1: Table A-5): articles were required to be written in English and birth prevalence was required to be based on confirmed cases. When two or more publications on any given region were identified, both were included if the research had been conducted by different groups, or if both the geography and time frame did not overlap. For reports with geographic and temporal overlap conducted by the same institution, the study covering the largest population was eligible.

### Data extraction and quality assessment

Extracted data elements included country and region, dates of data collection, study design, assay method for screening and for case confirmation (when diagnostic methods varied among sites or over time, scoring for the estimate was based on the lowest scoring diagnostic method, per the list in Table 2), diagnosis as reported in the publication (“nominal diagnosis”), Phe concentration used as a positive cutoff value, whether patients with BH4 deficiency were included in the number of cases reported, number of newborns screened, number of cases, and reported birth prevalence. For publications that reported birth prevalence stratified by multiple variables, values for each variable were extracted separately (herein referred to as “estimates”).

**Table 2:** Quality assessment tool for birth prevalence estimates

Scoring domain	Score		
	Strong	Moderate	Weak
Case definition^a^	The case definition is complete (including both screening positive and confirmed cases)	The case definition is partially complete (lacks either the definition of screening positive or of confirmed cases)	The case definition is incomplete for both screening positive and confirmed cases
Study setting/source population	Mandatory population-wide newborn screening program General population from a well-defined region and time	Catchment area of a hospital or other medical facility Hospital or laboratory records or disease registry Surveys (e.g., to health care providers)	Personal communication Unclear or not reported
Statistical methods	The denominator is the number of newborns screened, and cases in the numerator arise from the population in the denominator If any quantity is estimated rather than directly measured, estimations are in line with the criteria described here	The denominator is the overall number of births rather than the number screened	Cases in the numerator do not arise from the population in the denominator Unclear or not reported
Precision of prevalence estimate^b^	Half the width of the 95% confidence interval is less than half of the prevalence	Half the width of the 95% confidence interval is between half of the prevalence and the prevalence^c^	Half the width of the 95% confidence interval is greater than the prevalence Confidence interval is not estimable
Diagnostic method used for case confirmation^d^	Tandem mass spectrometry, high-performance liquid chromatography, column chromatography, (rapid) ion exchange chromatography, quantitative amino acid analyzer, positive mutational analysis, or enzymatic assay (including colorimetric, fluorimetric, and ELISA)	Guthrie test, bacterial inhibition assay, thin layer or paper chromatography	Other methods, or those where urine is used as the assay substrate Unclear or not reported

^a^The case definition was considered complete if the phenylalanine cutoff value was provided

^b^Additional file 1 presents the method of calculating the precision of the prevalence estimate

^c^Inclusive of both bounds

^d^When diagnostic methods varied among sites or over time, scoring for the estimate was based on the lowest scoring diagnostic method

Data were extracted by one researcher using a form specifically designed for this study; extracted data were verified by a second researcher. Each estimate was assessed for quality as strong, moderate, or weak in each of five scoring domains (Table 2). The quality assessment tool used in this study was based on existing tools for assessing the quality of studies that report the prevalence of conditions assessed by surveillance [22] or conditions of genetic origin [23].

### Meta-analyses

To mitigate errors that may arise from using early, less reliable assays, such as the Guthrie test, only estimates derived from confirmatory diagnostic assays that were assessed as strong in the quality assessment tool (Table 2) were eligible for meta-analysis. Inclusion in the meta-analysis also required that the number of cases and the number of screened newborns were reported. For each region and Phe concentration cutoff value category, at least 2 birth prevalence estimates were required to conduct a meta-analysis. For regions and Phe concentration cutoff value categories with only one published birth prevalence estimate, the single published estimate was used to represent the region (or Phe concentration cutoff value) in the global prevalence estimates. Once the eligible estimates for each planned meta-analysis were identified, estimates with both temporal and geographic overlap were assessed, and the estimate representing the largest geographic coverage or time period was included.

Meta-analyses were performed to determine aggregated regional birth prevalence (Europe, North America, Middle East/North Africa, Latin America, South Pacific, and West Pacific; Additional file 1: Table A-6) and a global birth prevalence. The global birth prevalence was estimated by using two approaches. A “regionally weighted” global prevalence was calculated, in which results from each region were weighted by the region’s relative numerical contribution to the total population of the regions for each analysis. For this determination, country-specific population counts were obtained from 2020 United Nations population estimates [24] and were summed within each region to determine regional totals (weights for analyses incorporating results from six regions: Europe, 0.126; Latin America, 0.097; Middle East/North Africa, 0.125; North America, 0.055; Southeast Asia, 0.303; West Pacific, 0.293). A non-regionally weighted global prevalence was also calculated for comparison to other recently published PKU global birth prevalence estimates that were not regionally-weighted.

For both regional and global birth prevalence determinations, birth prevalence was calculated and stratified by three confirmatory Phe concentration cutoff values (360 ± 100 μmol/L, 600 ± 100 μmol/L, 1200 ± 200 μmol/L). When a publication reported birth prevalence by Phe cutoff interval (e.g., separate birth prevalence values for ≥ 360 ± 100 to 600 μmol/L, ≥ 600 ± 100 μmol/L to 1200 μmol/L and ≥ 1200 ± 200 μmol/L), the sum of all values above the cutoff value was used. Finally, an unstratified meta-analysis was conducted, which additionally included estimates from studies in which Phe cutoff values were not reported, to determine overall (regionally weighted and non-regionally weighted) birth prevalence.

To provide appropriate weights for meta-analysis, birth prevalence estimates were transformed using the double arcsine method [25]; meta-analysis was conducted using a random-effects model with inverse variance weighting. Transformation and calculations were performed using MetaXL (version 5.3, EpiGear International). Heterogeneity was assessed using the I^*2*^ statistic [26, 27].

## Results

### Literature search and review

Searches in PubMed and Embase identified 1112 entries (Fig. 1). Screening of 997 unique PubMed and Embase entries and an additional 28 publications identified from reference lists of screened entries identified 85 publications meeting eligibility criteria, resulting in 238 birth prevalence estimates (Additional file 2).

These 85 publications were published from 1964 [28] to 2019 [29] and reported on data from 1960 [30] to 2018 [29] from 59 countries. Newborn blood or urine samples for screening were taken between the first day of life [31] and age 3–8 weeks [32]; 25 publications (125 birth prevalence estimates) did not report age at screening. Phe concentration used for confirmatory testing ranged from 120 μmol/L [33] to over 2600 μmol/L [34]. Forty-three publications (135 birth prevalence estimates) did not report the cutoff value for confirmatory testing. Nominal diagnoses were inconsistent. For example, classical PKU was defined using confirmatory Phe cutoff values ranging from 726 μmol/L [35] to 1816 μmol/L [36]. Cases with BH4 deficiency were included in 5 publications (6 birth prevalence estimates) and the presence or absence of BH4 deficiency was not reported in 58 publications (186 birth prevalence estimates).

**Fig. 2:**
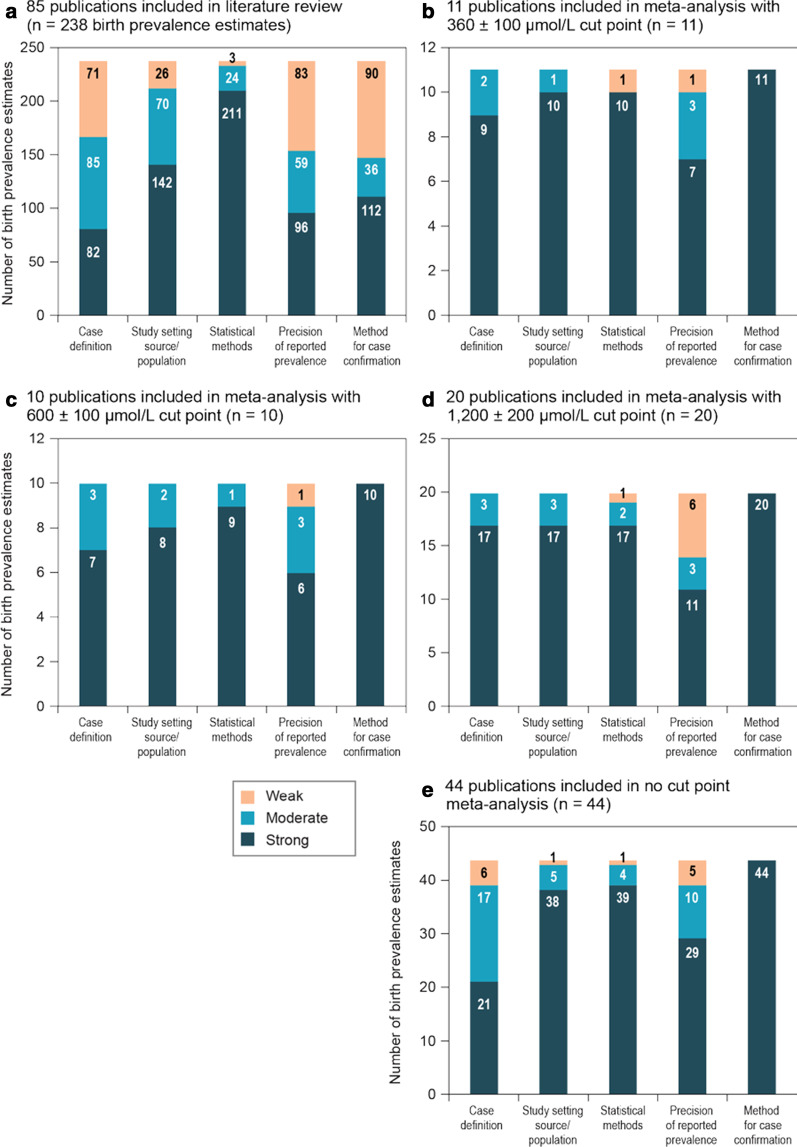
**a**–**e** Quality of evidence assessments of birth prevalence estimates

The only domains of the quality assessment tool on which > 50% of the estimates scored strong were *statistical methods* and *study setting/source population*. Sixty percent of the estimates scored moderate or weak on *precision*, and 53% scored moderate or weak on the *method for case confirmation* (Fig. 2A).

### Meta-analysis results

A total of 112 birth prevalence estimates (54 publications) scored strong in the quality assessment domain *diagnostic method used for case confirmation* and were therefore potentially eligible for meta-analysis. One publication (18 estimates) with strong scores in the *diagnostic method used for case confirmation* reported birth prevalence (in the format 1:8000), but did not provide the number of cases or screened newborns [37] and was not deemed eligible. No birth prevalence estimates from the African region were included in the meta-analysis, and the only estimates eligible for inclusion in Southeast Asia were from Thailand.

Birth prevalence estimates ranged from 0 (Estonia [38], Finland [39], and Thailand [40]) to 2.46 per 10,000 births (Macedonia) [41] (Table 3).

**Table 3:** Birth prevalence estimates scoring strong on diagnostic method for case confirmation (n = 54 publications)

Country	Birth prevalence per 10,000 newborns (95% CI)	Phe cutoff value for confirmatory diagnosis (µmol/L)	Score for additional quality of evidence domains^e^				References
			Case definition	Study setting source population	Statistical methods	Precision of prevalence estimate	
*Europe*							
Austria (Eastern, PKU^f^)	1.3 (0.92–1.83)^d^	NR	Weak	Strong	Strong	Strong	Thalhammer [49]
Austria (Eastern, HPA^f^)	0.49 (0.28–0.85)^d^	NR	Weak	Strong	Strong	Moderate	Thalhammer [49]
Austria (Western, PKU^f^)	0.45 (0.23–0.88)^d^	NR	Weak	Strong	Strong	Moderate	Thalhammer [49]
Austria (Western, HPA^f^)	0.5 (0.26–0.96)^d^	NR	Weak	Strong	Strong	Moderate	Thalhammer [49]
Estonia	0 (0–1.02)^d^	< 600	Strong	Strong	Strong	Weak	Ounap et al. [38]
Estonia	1.66 (0.76–3.63)^c,d^	1000	Strong	Strong	Strong	Moderate	Ounap et al. [38]
Finland	0 (0–0.52)^a,d^	363	Moderate	Strong	Strong	Weak	Visakorpi et al. [39]
Germany	0.81 (0.66–1)^d^	NR	Weak	Strong	Strong	Strong	Lindner et al. [50]
Germany	0.78 (0.63–0.97)^b,d^	600	Moderate	Strong	Strong	Strong	Lindner et al. [50]
Germany	0.81 (0.65–1.01)^a,d^	363– < 908	Strong	Strong	Strong	Strong	Mathias and Bickel [51]
Germany	0.99 (0.81–1.21)^a,d^	908	Strong	Strong	Strong	Strong	Mathias and Bickel [51]
Germany	0.96 (0.65–1.43)	600	Strong	Strong	Strong	Strong	Schulze et al. [52]
Germany	1.24 (0.87–1.76)	150–600	Strong	Strong	Strong	Strong	Schulze et al. [52]
Greece	0.44 (0.05–1.61)^d^	NR	Moderate	Moderate	Strong	Weak	Loukas et al. [53]
Greece	0.41 (0.31–0.56)^c,d^	1211	Strong	Strong	Strong	Strong	Missiou-Tsagaraki et al. (1988) [54]
Hungary	0.85 (0.39–1.86)^d^	NR	Weak	Strong	Strong	Moderate	Mehes et al. [55]
Italy (PKU^f^)	1.38 (0.85–2.23)^d^	NR	Moderate	Weak	Strong	Moderate	Antonozzi et al. [56]
Italy (HPA^f^)	0.26 (0.05–0.75)^d^	NR	Moderate	Weak	Strong	Weak	Antonozzi et al. [56]
Italy	0.22 (0.15–0.32)^c,d^	1211	Strong	Strong	Strong	Strong	Zaffanello et al. [57]
Italy	0.78 (0.63–0.96)^d^	NR	Moderate	Strong	Strong	Strong	Zaffanello et al. [57]
Macedonia	2.46 (0.06–13.68)^d^	151	Moderate	Strong	Strong	Weak	Kocova and Anastasovska [41]
Poland	0.28 (0.22–0.34)^a,d^	363–1211	Strong	Moderate	Strong	Strong	Cabalska et al. [58]
Poland	1.29 (1.16–1.42)^a,c,d^	1211	Strong	Moderate	Strong	Strong	Cabalska et al. [58]
Portugal	0.82 (0.56–1.2)^a,d^	360	Strong	Strong	Strong	Strong	Vilarinho et al. [59]
Portugal	0.38 (0.22–0.66)^d^	150–360	Strong	Strong	Strong	Moderate	Vilarinho et al. [59]
Slovakia	1.69 (1.45–1.98)^d^	NR	Weak	Strong	Strong	Strong	Dluholucký et al. [60]
Slovenia	0.98 (0.72–1.35)^b,c,d^	1200	Strong	Strong	Moderate	Strong	Smon et al. [61]
Slovenia	0.39 (0.24–0.64)^b,d^	600–900	Strong	Strong	Moderate	Moderate	Smon et al. [61]
Slovenia	0.1 (0.03–0.27)^b,d^	900–1200	Strong	Strong	Moderate	Weak	Smon et al. [61]
Spain	0.66 (0.22–1.55)^d^	240	Strong	Strong	Strong	Weak	Fernández-Iglesias et al. [62]
USSR/Russia	1.5 (0.98–2.3)^b,c,d^	1200	Moderate	Strong	Strong	Strong	Gerasimova et al. [63]
USSR/Russia	0.36 (0.12–0.84)^b,d^	600–1200	Moderate	Strong	Strong	Weak	Gerasimova et al. [63]
United Kingdom	0.49 (0.36–0.67)^c,d^	1200	Strong	Moderate	Strong	Strong	Walker et al. [64]
United Kingdom	0.19 (0.11–0.31)^d^	240	Strong	Moderate	Strong	Moderate	Walker et al. [64]
Yugoslavia	0.22 (0.1–0.48)^b,d^	605–902	Strong	Moderate	Strong	Moderate	Mardesic et al. [65]
Yugoslavia	0.69 (0.44–1.08)^b,d^	908	Strong	Moderate	Strong	Strong	Mardesic et al. [65]
*Latin America*							
Brazil (Laboratory A, 2005)	0.36 (0.12–0.84)^b,d^	605	Strong	Strong	Strong	Weak	Botler et al. [66]
Brazil (Laboratory A, 2006)	0.59 (0.31–1.12)^b,d^	605	Strong	Strong	Strong	Intermediate	Botler et al. [66]
Brazil (Laboratory A, 2007)	0.35 (0.11–0.82)^b,d^	605	Strong	Strong	Strong	Weak	Botler et al. [66]
Brazil (Laboratory B, 2005)	0.52 (0.06–1.9)^b,d^	605	Strong	Strong	Strong	Weak	Botler et al. [66]
Brazil (Laboratory B, 2006)	0.84 (0.17–2.45)^b,d^	605	Strong	Strong	Strong	Weak	Botler et al. [66]
Brazil (Laboratory B, 2007)	0.91 (0.11–3.28)^b,d^	605	Strong	Strong	Strong	Weak	Botler et al. [66]
Brazil	0.92 (0.25–2.36)^a–d^	1211	Strong	Strong	Strong	Weak	Ramalho et al. [67]
Brazil	0.23 (0.01–1.28)^a,d^	302–604	Strong	Strong	Strong	Weak	Ramalho et al. [67]
Brazil	0.23 (0.01–1.28)^a,b,d^	606–1210	Strong	Strong	Strong	Weak	Ramalho et al. [67]
Chile	0.53 (0.45–0.63)^c,d^	1211	Strong	Strong	Strong	Strong	Cornejo et al. [68]
Chile	0.98 (0.86–1.11)^d^	NR	Intermediate	Strong	Strong	Strong	Cornejo et al. [68]
*Middle East/North Africa*							
Iran	0.15 (0.07–0.32)^b,c,d^	1211	Strong	Strong	Strong	Intermediate	Abbaskhanian et al. [69]
Iran	0.29 (0.17–0.52)^d^	121–605	Strong	Strong	Strong	Intermediate	Abbaskhanian et al. [69]
Iran	0.22 (0.12–0.42)^b,d^	606–1210	Strong	Strong	Strong	Intermediate	Abbaskhanian et al. [69]
Iran	1.6 (1.11–2.31)^a,d^	424	Strong	Strong	Strong	Strong	Habib et al. [44]
Iran	0.52 (0.14–1.33)^d^	121– < 1211	Strong	Strong	Strong	Weak	Karamifar et al. [70]
Iran	0.39 (0.08–1.14)^c,d^	1211	Strong	Strong	Strong	Weak	Karamifar et al. [70]
Iran	1.92 (1.53–2.41)^d^	NR	Intermediate	Strong	Intermediate	Strong	Motamedi et al. [71]
Saudi Arabia	0.68 (0.52–0.89)^d^	180	Strong	Strong	Strong	Strong	Alfadhel et al. [72]
Turkey (classical PKU^f^)	1.35 (1.18–1.54)^d^	NR	Weak	Strong	Strong	Strong	Ozalp et al. [73]
Turkey (mild PKU^f^)	0.64 (0.52–0.77)^d^	NR	Weak	Strong	Strong	Strong	Ozalp et al. [73]
Turkey (mild HPA^f^)	0.36 (0.28–0.47)^d^	NR	Weak	Strong	Strong	Strong	Ozalp et al. [73]
UAE	0.76 (0.57–0.99)^c,d^	1211	Strong	Strong	Strong	Strong	Al Hosani et al. [74]
*North America*							
Canada (Alberta)	0.50 (CI not estimable)	NR	Weak	Intermediate	Strong	Weak	Somers and Favreau [37]
Canada (Ontario, PKU^f^)	0.60 (CI not estimable)	NR	Weak	Intermediate	Strong	Weak	Somers and Favreau [37]
Canada (Ontario, HPA^f^)	0.29 (CI not estimable)	NR	Weak	Intermediate	Strong	Weak	Somers and Favreau [37]
US (NC)	0.08 (0.01–0.3)	157	Intermediate	Strong	Strong	Weak	Frazier et al. [42]
US (NC)	0.46 (0.26–0.82)	250	Intermediate	Strong	Strong	Intermediate	Frazier et al. [42]
US (NC)	0.52 (0.39–0.69)^a,d^	300	Intermediate	Strong	Strong	Strong	Frazier et al. [42]
US (NY)	0.1 (0.05–0.2)^d^	908– < 1211	Strong	Strong	Intermediate	Intermediate	Hansen et al. [75]
US (NY)	0.53 (0.39–0.72)^c,d^	1211	Strong	Strong	Intermediate	Strong	Hansen et al. [75]
US (NY)	0.14 (0.07–0.25)^d^	NR	Intermediate	Strong	Intermediate	Intermediate	Hansen et al. [75]
US (NY)	0.7 (0.52–0.93)^d^	NR	Intermediate	Strong	Intermediate	Strong	Kelly and Palombi [76]
US (MA)	1.04 (0.62–1.75)^d^	NR	Intermediate	Strong	Strong	Intermediate	Maccready and Hussey [28]
US (CT)	0.83 (CI not estimable)	NR	Weak	Intermediate	Strong	Weak	Somers and Favreau [37]
US (FL)	1.00 (CI not estimable)	NR	Weak	Intermediate	Strong	Weak	Somers and Favreau [37]
US (KS)	0.80 (CI not estimable)	NR	Weak	Intermediate	Strong	Weak	Somers and Favreau [37]
US (KY)	0.87 (CI not estimable)	NR	Weak	Intermediate	Strong	Weak	Somers and Favreau [37]
US (OK)	0.59 (CI not estimable)	NR	Weak	Intermediate	Strong	Weak	Somers and Favreau [37]
US (PA, PKU^f^)	0.78 (CI not estimable)	NR	Weak	Intermediate	Strong	Weak	Somers and Favreau [37]
US (PA, HPA^f^)	0.18 (CI not estimable)	NR	Weak	Intermediate	Strong	Weak	Somers and Favreau [37]
US (TX)	0.38 (CI not estimable)	NR	Weak	Intermediate	Strong	Weak	Somers and Favreau [37]
US (VA)	0.57 (CI not estimable)	NR	Weak	Intermediate	Strong	Weak	Somers and Favreau [37]
US (WV)	0.67 (CI not estimable)	NR	Weak	Intermediate	Strong	Weak	Somers and Favreau [37]
US (PA)	0.43 (0.29–0.64)^a,d^	363	Strong	Strong	Strong	Strong	Wainer and Sideman [43]
US (PA)	0.9 (0.68–1.19)^d^	NR	Intermediate	Strong	Strong	Strong	Wainer and Sideman [43]
US (New England)	0.27 (0.13–0.56)^d^	NR	Intermediate	Strong	Strong	Intermediate	Zytkovicz et al. [77]
US (New England)	0.43 (0.24–0.77)^d^	NR	Intermediate	Strong	Strong	Intermediate	Zytkovicz et al. [77]
*Southeast Asia*							
Thailand	0.04 (0.01–0.08)	NR	Intermediate	Strong	Strong	Weak	Charoensiriwatana et al. [78]
Thailand	0.05 (0.02–0.11)	1211	Strong	Strong	Strong	Weak	Pangkanon et al. [79]
Thailand	0.03 (0.02–0.05)^c,d^	1200	Intermediate	Strong	Strong	Intermediate	Pangkanon et al. [80]
Thailand	0 (0–2.12)	NR	Intermediate	Intermediate	Strong	Weak	Ratrisawadi et al. [40]
Thailand	0.04 (0.01–0.1)	NR	Intermediate	Strong	Strong	Weak	Sutivijit et al. [81]
*West Pacific*							
Australia	0.26 (0.09–0.61)^d^	200–300	Intermediate	Intermediate	Strong	Weak	Boneh et al. [34]
Australia	0.37 (0.18–0.76)^b,d^	600–1200	Intermediate	Intermediate	Strong	Intermediate	Boneh et al. [34]
Australia	0.05 (0–0.29)^b,d^	2600	Intermediate	Intermediate	Strong	Weak	Boneh et al. [34]
China	0.17 (0.08–0.36)	242–1211	Strong	Strong	Strong	Intermediate	Chen et al. [82]
China	0.59 (0.38–0.89)^c^	1211	Strong	Strong	Strong	Strong	Chen et al. [82]
China	0.38 (0.23–0.64)^d^	NR	Weak	Strong	Strong	Intermediate	Lin et al. [29]
China	0.1 (0.01–0.36)^a,d^	363– < 908	Strong	Strong	Strong	Weak	Liu and Zuo [83]
China	0.5 (0.27–0.93)^a,d^	908 or 1211	Strong	Strong	Strong	Intermediate	Liu and Zuo [83]
China	0.4 (0.34–0.47)	NR	Intermediate	Strong	Strong	Strong	Maitusong et al. [84]
China	0.91 (0.65–1.28)	NR	Weak	Intermediate	Strong	Strong	Shi et al. [85]
China	0.65 (0.48–0.9)^a,c,d^	1200	Strong	Strong	Weak	Strong	Su et al. [86]
China	0.28 (0.17–0.45)^d^	120–360	Strong	Strong	Weak	Intermediate	Su et al. [86]
China	0.98 (0.76–1.27)^a,d^	360–1200	Strong	Strong	Weak	Strong	Su et al. [86]
China	0.88 (0.46–1.67)	NR	Weak	Strong	Strong	Intermediate	Tu et al. [87]
China	0.07 (0.01–0.21)^a–d^	1200	Strong	Strong	Strong	Weak	Wang et al. [88]
China	0.1 (0.03–0.24)^d^	120–360	Strong	Strong	Strong	Weak	Wang et al. [88]
China	0.05 (0.01–0.17)^a,d^	360–600	Strong	Strong	Strong	Weak	Wang et al. [88]
China	0.14 (0.07–0.31)^a,b,d^	600–1200	Strong	Strong	Strong	Intermediate	Wang et al. [88]
China	0.86 (0.82–0.91)^d^	NR	Intermediate	Strong	Strong	Strong	Zhan et al. [89]
South Korea	0.51 (0.14–1.29)^c,d^	1200	Strong	Strong	Strong	Weak	Yoon et al. [90]
Taiwan	0.27 (0.2–0.36)^d^	120– < 600	Strong	Strong	Strong	Strong	Niu et al. [91]
Taiwan	0.13 (0.09–0.21)^b,d^	600– < 1200	Strong	Strong	Strong	Strong	Niu et al. [91]
Taiwan	0.03 (0.01–0.08)^b–d^	1200	Strong	Strong	Strong	Weak	Niu et al. [91]

CI = confidence interval; CT = Connecticut; FL = Florida; HPA = hyperphenylalaninemia; KS = Kansas; KY = Kentucky; MA = Massachusetts; NC = North Carolina; NR = not reported; NY = New York; OK = Oklahoma; PA = Pennsylvania; Phe = phenylalanine; PKU = phenylketonuria; TX = Texas; UAE = United Arab Emirates; US = United States; USSR = Union of Soviet Socialist Republics; VA = Virginia; WV = West Virginia

^a^Estimate contributes to meta-analysis with diagnostic cutoff value 360 µmol/L

^b^Estimate contributes to meta-analysis with diagnostic cutoff value 600 µmol/L

^c^Estimate contributes to meta-analysis with diagnostic cutoff value 1200 µmol/L

^d^Estimate contributes to overall meta-analysis

^e^This table includes only estimates for which the method of diagnosis confirmation was considered “strong” in the quality of evidence scoring tool

^f^Nominal diagnoses as provided in associated reference

Estimates from 45 publications were included in at least one meta-analysis, and the rest were excluded due to temporal and regional overlap. Meta-analysis results are summarized in Table 4 and Additional file 3: Figures A2–A5. The regionally weighted global birth prevalence of PAH deficiency (N = 44 publications, 1 estimate per publication) was 0.64 (95% confidence interval [CI] 0.53–0.75) per 10,000 births (Table 4; quality assessment results shown in Fig. 2E). The lowest regional birth prevalence was observed in Southeast Asia, with 0.03 cases per 10,000 births (95% CI 0.02–0.05); the highest was observed in the Middle East/North Africa, with 1.18 (95% CI 0.64–1.87) cases per 10,000 births.

**Table 4:** Meta-analysis^a^ of birth prevalence estimates stratified by region and by phenylalanine diagnostic cutoff value

Region	Birth prevalence per 10,000 screened (95% CI)	*I*^2^ (%)	Number of estimates	Reference(s)	Country
*Confirmatory test phenylalanine cutoff value of 360* ± *100 µmol/L*					
Europe	0.97 (0.52–1.53)	93.8	4	Cabalska et al. [58]	Poland
				Mathias and Bickel [51]	Germany
				Vilarinho et al. [59]	Portugal
				Visakorpi et al. [39]	Finland
Latin America	1.38 (0.51–3.01)	NA	1	Ramalho et al. [67]	Brazil
Middle East/North Africa	1.60 (1.06–2.31)	NA	1	Habib et al. [44]	Iran
North America	0.49 (0.38–0.61)	0.0	2	Frazier et al. [42]	United States
				Wainer and Sideman[43]	United States
West Pacific	0.63 (0.03–1.75)	96.5	3	Liu and Zuo [83]	China
				Su et al. [86]	China
				Wang et al. [88]	China
Global (non-regionally weighted)	0.85 (0.51–1.26)	95.9	11	–	–
Global (regionally weighted)^b^	0.96 (0.50–1.42)	NA	11	–	–
*Confirmatory test phenylalanine cutoff value of 600* ± *100 µmol/L*					
Europe	1.18 (0.75–1.69)	85.8	4	Lindner et al. [50]	Germany
				Gerasimova et al. [63]	USSR/Russia
				Mardesic et al. [65]	Yugoslavia
				Smon et al. [61]	Slovenia
Latin America	0.65 (0.14–1.46)	64.2	2	Botler et al. [66]	Brazil
				Ramalho et al. [67]	Brazil
Middle East/North Africa	0.37 (0.21–0.61)	NA	1	Abbaskhanian et al. [69]	Iran
West Pacific	0.23 (0.12–0.36)	55.9	3	Boneh et al. [34]	Australia
				Niu et al. [91]	Taiwan
				Wang et al. [88]	China
Global (non-regionally weighted)	0.66 (0.38–1.02)	94.1	10	–	–
Global (regionally weighted)^b^	0.50 (0.37–0.64)	NA	10	–	–
*Confirmatory test phenylalanine cutoff value of 1200* ± *200 µmol/L*					
Europe	0.78 (0.40–1.3)	96.9	7	Cabalska et al. [58]	Poland
				Gerasimova et al. [63]	USSR/Russia
				Missiou-Tsagaraki et al. [54]	Greece
				Ounap et al. [38]	Estonia
				Smon et al. [61]	Slovenia
				Walker et al. [64]	United Kingdom
				Zaffanello et al. [57]	Italy
Latin America	0.58 (0.30–0.94)	29.2	2	Cornejo et al. [68]	Chile
				Ramalho et al. [67]	Brazil
Middle East/North Africa	0.36 (0.04–0.94)	91.2	3	Abbaskhanian et al. [69]	Iran
				Karamifar et al. [70]	Iran
				Al Hosani et al. [74]	United Arab Emirates
North America	0.53 (0.38–0.72)	NA	1	Hansen et al. [75]	United States
Southeast Asia	0.03 (0.02–0.05)	NA	1	Pangkanon et al. [80]	Thailand
West Pacific	0.22 (0.03–0.56)	94.6	6	Boneh et al. [34]	Australia
				Chen et al. [82]	China
				Niu et al.[91]	Taiwan
				Su et al. [86]	China
				Yoon et al. [90]	South Korea
				Wang (2019) [88]	China
Global (non-regionally weighted)	0.47 (0.26–0.74)	98.0	20	–	–
Global (regionally weighted)^b^	0.30 (0.20–0.40)	NA	20	–	–
*Overall analysis*^*c*^					
Europe	1.14 (0.89–1.41)	92.2	19	Antonozzi et al. [56]	Italy
				Cabalska et al. [58]	Poland
				Dluholucký and Knapková [60]	Slovakia
				Fernández-Iglesias et al. [62]	Spain
				Gerasimova et al. [63]	USSR/Russia
				Kocova and Anastasovska [41]	Macedonia
				Lindner et al. [50]	Germany
				Loukas et al. [53]	Greece
				Mardesic et al. [65]	Yugoslavia
				Mathias and Bickel [51]	Germany
				Mehes et al. [55]	Hungary
				Missiou-Tsagaraki et al. [54]	Greece
				Ounap et al. [38]	Estonia
				Smon et al. [61]	Slovenia
				Thalhammer [49]	Austria
				Vilarinho et al. [59]	Portugal
				Visakorpi et al. [39]	Finland
				Walker et al. [64]	United Kingdom
				Zaffanello et al. [57]	Italy
Latin America	0.98 (0.29–2.03)	95.8	3	Botler et al. [66]	Brazil
				Cornejo et al. [68]	Chile
				Ramalho et al. [67]	Brazil
Middle East/North Africa	1.18 (0.64–1.87)	96.5	7	Abbaskhanian et al. [69]	Iran
				Alfadhel et al. [72]	Saudi Arabia
				Al Hosani et al. [74]	United Arab Emirates
				Habib et al. [44]	Iran
				Karamifar et al. [70]	Iran
				Motamedi et al. [71]	Iran
				Ozalp et al. [73]	Turkey
North America	0.81 (0.58–1.07)	82.3	6	Frazier et al. [42]	United States
				Hansen et al. [75]	United States
				Kelly and Palombi [76]	United States
				Maccready and Hussey [28]	United States
				Wainer and Sideman [43]	United States
				Zytkovicz et al. [77]	United States
Southeast Asia	0.03 (0.02–0.05)	NA	1	Pangkanon et al. [80]	Thailand
West Pacific	0.68 (0.43–0.98)	94.2	8	Boneh et al. [34]	Australia
				Lin et al. [29]	China
				Liu and Zuo [83]	China
				Niu et al. [91]	Taiwan
				Su et al. [86]	China
				Wang et al. [88]	China
				Yoon et al. [90]	South Korea
				Zhan et al. [89]	China
Global (non-regionally weighted)	0.96 (0.75–1.19)	98.0	44	–	–
Global (regionally weighted)^b^	0.64 (0.53–0.75)	NA	44	–	–

CI = confidence interval; NA = not available

^a^Includes only estimates in which the diagnostic method used for case confirmation was considered strong in the quality assessment tool

^b^Global prevalence was calculated by weighting each region by its relative contribution to the total population

^c^Includes estimates for which the diagnostic cutoff value was not reported. When a publication reported birth prevalence by Phe cutoff intervals, the value used was for the sum of the intervals

Eleven publications reported birth prevalence estimates (1 estimate per publication) with a confirmatory test Phe concentration cutoff value of 360 ± 100 µmol/L. The regionally weighted global birth prevalence was 0.96 (95% CI 0.50–1.42) per 10,000 births (Table 4 and Fig. 2B). The lowest regional birth prevalence was observed in North America, with 0.49 cases per 10,000 births (95% CI 0.38–0.61), based on two publications that presented very similar results [42, 43], as reflected in the heterogeneity statistic *I*^2^ value of 0. The highest birth prevalence was observed in the Middle East/North Africa, 1.60 (95% CI 1.06–2.31) per 10,000 births, based on a single estimate [44].

Ten publications (1 estimate each) reported birth prevalence estimates using a confirmatory test Phe concentration cutoff value of 600 ± 100 µmol/L. The regionally weighted global birth prevalence was 0.50 (95% CI 0.37–0.64) per 10,000 births (Table 4 and Fig. 2C) for this cutoff value.

For the 1200 ± 200 µmol/L cutoff value for a Phe concentration confirmatory test, 20 publications (1 estimate each) were eligible and the regionally weighted global birth prevalence was 0.30 (95% CI 0.20–0.40) per 10,000 births (Table 4 and Fig. 2D).

## Discussion

The overall meta-analysis conducted in this systematic review provides a regionally weighted global birth prevalence of PAH deficiency of 0.64 (95% CI 0.53–0.75) per 10,000 births. It is important to weight birth prevalence estimates by region so that the global PAH deficiency birth prevalence reflects both the birth prevalence and population size of each region rather than just the inverse variance (primarily driven by the sample size) of the individual studies (as was done for the calculation of non–regionally weighted birth prevalence). The highest regional birth prevalence in the overall analysis was reported in the Middle East/North Africa, where consanguineous marriages are among the most frequent in the world, with frequencies up to 42% in Saudi Arabia [45].

Among estimates with a confirmatory test Phe concentration cutoff value of 360 ± 100 µmol/L, the regionally weighted global birth prevalence was 0.96 (95% CI 0.50–1.42) per 10,000 births. On the basis of recent European and American College of Medical Genetics and Genomics guidelines (Table 1), this would represent the population for which treatment in children is recommended. Based on the single estimate for Middle East/North Africa, the birth prevalence was again highest in this region [44].

In the meta-analyses based on Phe concentration cutoff values of 600 µmol/L and 1200 µmol/L, the regionally weighted global prevalences were 0.50 (95% CI 0.37–0.64) and 0.30 (95% CI 0.20–0.40), respectively, per 10,000 births. Regional variation in the prevalence of PAH deficiency defined by these cutoff values was observed, with higher prevalences in Europe, Latin America, North America, and the Middle East than was observed globally. In a recent analysis of global variations in PAH genotype [46], genotypes associated with classical PKU (Phe ≥ 1200 µmol/L) tended to be the most common in the Middle East.

As might be expected, in this meta-analysis we observed decreasing pooled birth prevalence as confirmatory test Phe cutoff values increased (Table 4). The decreasing prevalence we observed with increasing Phe cutoff values should be interpreted cautiously. Specifically, this finding does not necessarily reflect differences in the relative frequencies of classical, moderate, mild PKU and HPA, but rather the fact that individuals with higher Phe levels are included in the estimates with lower cutoff values (e.g., the pooled prevalence for the 360 µmol/L cutoff value includes individuals that would be diagnosed as having classical and severe PKU per Table 1). This approach was taken to ascertain the birth prevalence of all individuals whose Phe levels were within the treatable range and the impact different confirmatory Phe cutoff thresholds have on PAH deficiency birth prevalence estimates. The confidence intervals for the various Phe cutoff thresholds had substantial overlap, likely due to heterogeneity of estimates from individual studies.

As evidenced by the high *I*^2^ values, heterogeneity of birth prevalence estimates was generally high, even among estimates stratified by region and Phe concentration cutoff values for case confirmation. Heterogeneity may be partly explained by random variation related to sampling, which is supported by the fact that many included studies were small (35% of the 238 estimates scored weak on *precision of the prevalence estimate* [Fig. 2]). Other reasons for heterogeneity include variations in age at screening and confirmatory testing, and dietary intake prior to sampling.

We found that data elements that are key to understanding the reported birth prevalence estimates were often missing: 30% of the 238 estimates scored weak on *case definition* (i.e., failed to provide Phe cutoff values for both screening and for case confirmation), and 66% scored moderate on this domain (failed to report on either screening or confirmatory Phe cutoff values); 11% did not report the *study setting/source population* or derived the information from personal communications. In addition, 126 of 238 reported birth prevalence estimates (53%) scored moderate or weak in *diagnostic method used for case confirmation*. Thirteen percent of the 238 estimates lacked information on the time period assessed, 3% on the assay used for screening, and 38% on the assay used for case confirmation. Although the frequency of BH4 deficiency is very low (1–2% of HPA cases) [6], it was not reported or not excluded from the reported birth prevalence estimates in 81% of the 238 birth prevalence estimates included in this review.

Substantial inconsistencies were observed in the nominal diagnoses reported, even in recent publications, with poor or inaccurate distinction between PKU, moderate PKU, classical PKU, and HPA (Additional file 2).

We have not found published papers estimating the global prevalence of PAH deficiency. However, two recently published reviews estimated the global prevalence of PKU. Shoraka et al. [47] identified studies reporting the birth prevalence of classical PKU in newborns and meta-analyzed them by region and overall (non-regionally weighted, with no stratification by case confirmation Phe cut off value). Hillert et al. [46] used unpublished information from national screening centers and reports identified through a literature search to estimate a global prevalence of PKU in newborns. Table 5 provides a comparison of the birth prevalence estimates from our analysis with the results from the studies by Shoraka et al. [46] and Hillert et al. [45].

**Table 5:** Comparison of birth prevalence estimates among recent literature reviews

Region	Birth prevalence estimate per 10,000 (95% CI)		
	Hillert et al. [46]	Shoraka et al. [47]	This study^b^
Europe^a^	NR	0.81 (0.65–0.97)	1.14 (0.89–1.41)
Middle East/North Africa	NR	NR	1.18 (0.64–1.87)
Eastern Mediterranean	NR	0.98 (0.62–1.35)	NR
Pan America	NR	0.53 (0.46–0.61)	NR
Latin America	NR	NR	0.98 (0.29–2.03)
North America	NR	NR	0.81 (0.58–1.07)
Southeast Asia	NR	0.03 (0.02–0.05)	0.03 (0.02–0.05)
West Pacific	NR	0.29 (0.09–0.50)	0.68 (0.43–0.98)
Global (non-regionally weighted)	NR	0.60 (0.51–0.69)	0.96 (0.75–1.19)
Global (regionally weighted)	0.42 (NR)	NR	0.64 (0.53–0.75)^c^

CI = confidence interval; NR = not reported

^a^Shoraka et al. incorrectly classified one included publication as European when it was in fact a North American study

^b^Table 4 presents the birth prevalence estimates from this analysis in further detail

^c^Global prevalence was calculated by weighting each region by its relative contribution to the total population

The largest differences between the current study and the study by Shoraka et al. were seen in Europe, the Americas, and the West Pacific regions. The similarity between the overall estimate by Shoraka et al. and the currently reported regionally weighted global birth prevalence is likely largely due to chance, as substantially different inclusion criteria and methodologies were employed in the two studies (Additional file 1: Figure A-1). Shoraka et al. excluded publications considered to have a high risk of bias as assessed using an existing 10-point checklist [48], which has some similar elements to the quality of evidence tool used in this publication. There was no requirement that cases be confirmed. The reported prevalence was described as relating to classical PKU, even though the Phe cutoff for confirmatory tests of the included studies ranged from 1.65 mg/dL (equivalent to 100 µmol/L) to 20 mg/dL (1211 µmol/L).

The current study provides a higher estimate of the global birth prevalence of PAH deficiency than Hillert et al. Unfortunately the inclusion and exclusion criteria and the method(s) for combining estimates from individual studies are not fully described in that paper, nor are the sources fully described; the global estimate included data from countries that the study describes as lacking newborn screening programs in parts of Africa, Asia, South America, and the Caribbean [46].

The current findings confirm that regional differences exist in the birth prevalence of PAH deficiency, with higher frequencies of inheritance of this autosomal recessive disease in areas with higher frequencies of consanguineous marriages, as has also been noted by others [46, 47].

Limitations of this study include incomplete reporting of key data elements in many of the included publications. In addition, the precision of the reported prevalence was low for most of the included estimates due to small sample sizes. No articles were identified reporting on the birth prevalence of PAH deficiency in Sub-Saharan Africa and birth prevalence estimates from countries in Southeast Asia were limited, lacking representation of some of the most populous countries in the region such as India. Absence of estimates could be attributed to absence of newborn screening programs for PAH deficiency in specific countries and regions [15], or lack of published estimates from newborn screening programs meeting the inclusion criteria for this review, such as the requirement that the full-text article be written in English. Strengths of this study include the fact that only confirmed cases were included in the qualitative synthesis, and that the meta-analysis only included estimates based on higher quality confirmatory assays. In addition, meta-analyses were undertaken based on clinically relevant diagnostic cutoff values.

## Conclusions

In this systematic literature review and meta-analysis, we estimated the regionally weighted global birth prevalence of PAH deficiency to be 0.64 (95% CI 0.53–0.75) per 10,000 births (overall). The estimated regionally weighted global birth prevalence among newborns with Phe level ≥ 360 ± 100 µmol/L at diagnosis was 0.96 (95% CI 0.50–1.42), which is the population for whom treatment is recommended. Substantial regional variation was observed with an elevated birth prevalence of this autosomal recessive disease in regions with higher frequencies of consanguineous births. Despite the fact that newborn screening has been widely implemented in much of the world for decades, the precision of the estimates is limited by the unavailability of publications on large population samples. This observation underscores the need for more comprehensive and systematic data collection as well as improved standards for reporting results. Only with more widespread availability of data from newborn screening programs from large populations will it be possible to obtain robust estimates and truly understand the magnitude of this serious and treatable condition.

## Supplementary Information

**Additional file 1.** Literature search strategy, quality assessment, and country region classification. Description of the literature search strategy, how quality assessments were calculated for precision, and how country regions were classified.

**Additional file 2.** Data extraction table. 238 birth prevalence estimates from 85 publications.

**Additional file 3.** Meta-analysis forest plot figures. **Figure A-2**. Meta-Analysis Results by Region: Confirmatory Test Phenylalanine Cutoff Value of 360 ± 100 μmol/L. **Figure A-3**. Meta-Analysis Results by Region: Confirmatory Test Phenylalanine Cutoff Value of 600 ± 100 μM/L. **Figure A-4**. Meta-Analysis Results by Region: Confirmatory Test Phenylalanine Cutoff Value of 1200 ± 200 μM/L. **Figure A-5**. Meta-Analysis Results by Region: Overall Analysis.

## Data Availability

The dataset supporting the conclusions of this article is within the published manuscript and its appendices.

## Abbreviations

ACMG: American College of Medical Genetics and Genomics
BH4: Tetrahydrobiopterin
CI: Confidence interval
HPA: Hyperphenylalaninemia
NA: Not available
NR: Not reported
PAH: Phenylalanine hydroxylase
Phe: Phenylalanine
PICOS: Population, intervention, comparison, outcomes, study design
PKU: Phenylketonuria
US: United States
