# Supplementary material for: Birth prevalence of phenylalanine hydroxylase deficiency: a systematic literature review and meta-analysis
Source: Orphanet J Rare Dis. 2021 Jun 3;16:253. doi: 10.1186/s13023-021-01874-6 (PMC8173927; doi:10.1186/s13023-021-01874-6)
Supplement: Supplementary file 1 — Additional file 1. Literature search strategy, quality assessment, and country region classification. Description of the literature search strategy, how quality assessments were calculated for precision, and how country regions were classified. [file 13023_2021_1874_MOESM1_ESM.pdf]

ADDITIONAL FILE 1

1. Literature search strategy and eligibility criteria

Table A-1. Research Question in PICOS Framework

PICOS Headings	Content
Population	Newborns from unselected human populations
Intervention (or exposure)	Prevalence, incidence, epidemiology, screening
Comparison	Not applicable (the objective of this study is descriptive rather than comparative)
Outcomes	Confirmed phenylketonuria identified from biological samples
Study design	Original research published in any language (English-language abstract required)

PICOS = population, intervention, comparison, outcomes, study design.

Table A-2. PubMed Search Terms

#	PICOS Headings	Description	Search Terms
1	Population	Population	("Infant, Newborn"[Mesh] OR newborn*[Title] OR neonate*[Title])
2	Intervention	Prevalence	("Prevalence"[Majr] OR prevalen*[Title/Abstract])
3		Incidence	("Incidence"[Majr] OR inciden*[Title/Abstract])
4		Epidemiology	("Phenylketonurias/epidemiology"[Majr] OR "Epidemiology"[Majr] OR epidemiol*[Title/Abstract])
5		Screening	("Phenylketonurias/diagnosis"[Majr] OR "Diagnosis"[Majr] OR "Neonatal Screening"[Majr] OR screen*[Title] OR diagnos*[Title] OR surveillance[Title] OR "Guthrie test"[Title/Abstract] OR heel[Title/Abstract] OR blood spot*[Title/Abstract] OR "dry blood"[Title/Abstract] OR "dried blood"[Title/Abstract])
	Comparison		Not applicable
6	Outcomes	Phenylketonuria	"Phenylketonurias"[Majr] OR phenylketonuria*[Title] OR hyperphenylalaninemia*[Title] OR "phenylalanine hydroxylase deficiency"[Title] OR PKU[Title]
7	Study design	Type of publication (for exclusion)	"Comment"[Publication Type] OR "Letter"[Publication Type] OR "Editorial"[Publication Type
8		Non-human research (for exclusion)	"Animals"[Mesh] NOT "Humans"[Mesh]

PICOS = population, intervention, comparison, outcomes, study design.

Note: The search was implemented as #1 and #6 and (#2 or #3 or #4 or #5) not (#7 or #8), on October 14, 2019, sorted by best match.

Table A-3. Embase Search Terms

#	PICOS Headings	Description	Search Terms
1	Population	Population	('newborn'/exp OR newborn*:ti OR neonate*:ti)
2	Intervention	Prevalence	('prevalence'/exp/mj OR prevalen*:ti,ab)
3		Incidence	('incidence'/exp/mj OR inciden*:ti,ab)
4		Epidemiology	('phenylketonuria'/exp/mj/dm_ep OR 'epidemiology'/exp/mj OR epidemiol*:ti,ab)
5		Screening	('phenylketonuria'/exp/mj/dm_di OR 'diagnosis'/exp/mj OR 'newborn screening'/exp/mj OR screen*:ti OR diagnos*:ti OR surveillance:ti OR 'Guthrie test':ti,ab OR heel:ti,ab OR (blood NEXT/1 spot*):ti,ab OR 'dry blood':ti,ab OR 'dried blood':ti,ab)
	Comparison		Not applicable
6	Outcomes	Phenylketonuria	'phenylketonuria'/exp/mj OR phenylketonuria*:ti OR hyperphenylalaninemia*:ti OR 'phenylalanine hydroxylase deficiency':ti OR 'PKU':ti
7	Study design	Type of publication (for exclusion)	comment*:ti OR letter:it OR editorial:it OR [conference abstract]/lim OR [conference paper]/lim OR "conference abstract":it OR "conference paper":it OR 'conference proceeding':pt
8		Non-human research (for exclusion)	'animal'/exp NOT 'human'/exp

PICOS = population, intervention, comparison, outcomes, study design.

Note: The search was implemented as #1 and #6 and (#2 or #3 or #4 or #5) not (#7 or #8), on October 14, 2019.

Table A-4. Study Eligibility Criteria for Level 1 Screening

- Conference abstracts were not eligible.
- The abstract, if present, reflected that the paper presented original research (relevant reviews were marked for future reference). In publications without abstract, the title indicated that the publication presents original research.
- The abstract contained numeric reports on the birth prevalence of PKU in newborns (e.g., "1 per 10,000 screened infants were confirmed as having PKU"), contained the information to calculate the prevalence (e.g., "of 8,530 screened infants, 2 were confirmed as PKU cases"), or contained text reflecting that such information was presented in the full text (e.g., "We report the birth prevalence of PKU in country X") or, in publications without an abstract, the title indicated that such numbers would be presented in the full text.
- The prevalence or incidence reported was in an unselected population (e.g., a report on the prevalence of PKU in children hospitalized in a psychiatric institution was not eligible).
- The prevalence or incidence reported was based on tests conducted on biological samples (estimates from models were not eligible).
- Studies that reported exclusively on BH4 deficiency (and did not report on PKU deficiency) were not eligible.
- Studies focusing on developing or validating assays or methodology were not eligible (even if they provided prevalence estimates).
- When duplicate entries were identified that met criteria to go into level 2 screening, one of the entries was be excluded. If one entry was in English and one was in a different language, only the entry in English was retained.

BH4 = tetrahydrobiopterin; PKU = phenylketonuria.

Table A-5. Additional Study Eligibility Criteria for Level 2 Screening

- The study reported on <u>confirmed</u> cases of PKU
- The full-text article was written in English
- If there were two or more reports on any given country or region (e.g., birth prevalence of PKU in the USA in years 1980-2000 and in Massachusetts in years 2010-2015), the following process will apply:
- If there was no geographic or temporal overlap, both studies were included
- If there was geographic and temporal overlap, and the studies were conducted by the same institution, the study covering the largest population was included
- If there was geographic and temporal overlap and the studies had been conducted by different institutions, all studies were eligible for data extraction (not for meta-analysis)
- If questions remained, the research team discussed to reach consensus on which study(-ies) should be included

PKU = phenylketonuria; US = United States of America.

2. Calculation of precision for quality assessment

One of the domains of the quality assessment tool examined the precision of the estimated prevalence by comparing the prevalence with the width of the 95% confidence interval. The categories for this domain are:

- Half the width of the 95% confidence interval is less than half of the prevalence (considered strong).
- Half the width of the 95% confidence interval is between half of the prevalence and the prevalence (moderate).
- Half the width of the 95% confidence interval is greater than the prevalence (weak).
- Confidence interval is not estimable (weak).

To facilitate the assessment of this criterion, calculations were embedded in the data extraction table.

Calculations for the 95% confidence interval were as follows:

- $n \times p$ was calculated as the number of screened newborns multiplied by the prevalence
- $n \times (1 - p)$ was calculated as the number of screened newborns multiplied by (1 minus the prevalence)
- If both $n \times p$ and $n \times (1 - p)$ were larger than 5, a normal approximation was used to estimate the 95% confidence interval [1]
- Otherwise, Fisher's exact approach was used in an implementation for a worksheet [2].

3. Classification of countries into regions

Classification into geographic regions was based on the World Health Organization (WHO) classification [3]. Changes to this classification implemented for this study were the addition of countries not included in the current version of the classification (e.g., England, Taiwan, and Yugoslavia), division of Pan America into North America and Latin America. Israel and Turkey were reassigned from the European region into the Eastern Mediterranean region, based on considerations of consanguinity patterns and geography. Group labels were slightly modified for clarity. Countries with available data were included in each region as listed in Table A-6.

Table A-6. Classification of Countries Into Regions for 59 Countries (85 Studies) Included in This Literature Review

Region	Countries
Europe	Austria, Belarus, Belgium, Bosnia-Herzegovina, Bulgaria, Croatia, Cyprus, Czech Republic, Denmark, East Germany, England, Estonia, Finland, France, Germany, Greece, Hungary, Iceland, Ireland, Italy, Latvia, Lithuania, Luxembourg, Macedonia, Netherlands, Northern Ireland, Norway, Poland, Portugal, Russia, Scotland, Serbia, Slovakia, Slovenia, Spain, Sweden, Switzerland, USSR/Russia, Ukraine, United Kingdom, Wales, Yugoslavia
Latin America	Brazil, Chile
Middle East/North Africa	Egypt, Iran, Israel, Saudi Arabia, Turkey, United Arab Emirates
North America	Canada, United States of America
South East Asia	Thailand
West Pacific	Australia, China, Japan, New Zealand, South Korea, Taiwan

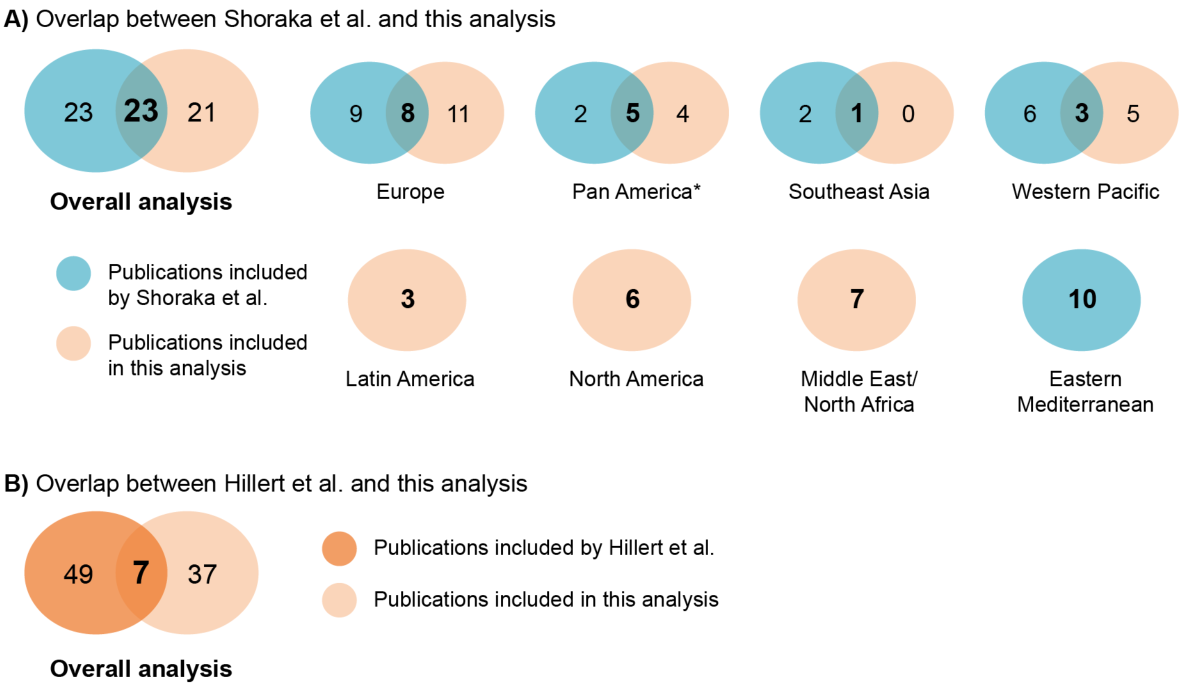

* In the current study, North America and Latin America were studied separately.

Figure A-1. Overlap of studies included in recent literature reviews

Additional file references

[1] Rothman KJ. Episheet—spreadsheets for the analysis of epidemiologic data, tab Quickcalc. 2015. Available at: http://www.krothman.org/episheet.xls. Accessed: October 11, 2019.

[2] Pezullo J. Exact binomial and poisson confidence intervals. 25 May 2009. Available at: https://statpages.info/confint.html; https://statpages.info/confint.xls (tab Another approach).

[3] WHO. Alphabetical List of WHO Member States. Available at: https://www.who.int/choice/demography/by_country/en/. Accessed: 7 Sep 2020.
