# Supplementary material for: Birth prevalence of phenylalanine hydroxylase deficiency: a systematic literature review and meta-analysis
Source: Orphanet J Rare Dis. 2021 Jun 3;16:253. doi: 10.1186/s13023-021-01874-6 (PMC8173927; doi:10.1186/s13023-021-01874-6)
Supplement: Supplementary file 3 — Additional file 3. Meta-analysis forest plot figures. Figure A-2. Meta-Analysis Results by Region: Confirmatory Test Phenylalanine Cutoff Value of 360 ± 100 μmol/L. Figure A-3. Meta-Analysis Results by Region: Confirmatory Test Phenylalanine Cutoff Value of 600 ± 100 μM/L. Figure A-4. Meta-Analysis Results by Region: Confirmatory Test Phenylalanine Cutoff Value of 1200 ± 200 μM/L. Figure A-5. Meta-Analysis Results by Region: Overall Analysis. [file 13023_2021_1874_MOESM3_ESM.pdf]

ADDITIONAL FILE 3

Figure A-2. Meta-Analysis Results by Region: Confirmatory Test Phenylalanine Cutoff Value of 360 ± 100 μmol/L

Study	Cases	Screened newborns	Birth prevalence per 10,000 screened newborns (95% CI)	Weight
**Region = Europe**				
Visakorpi (1971), Finland	0	71,135	0.00 (0.00-0.52)	7.6%
Mathias (1986), Germany	169	940,369	1.80 (1.54-2.09)	9.8%
Cabalska (1993), Poland	447	2,861,504	1.56 (1.42-1.71)	10.0%
Vilarinho (2010), Portugal	26	316,243	0.82 (0.54-1.20)	9.4%
**Overall, Europe**		**4,189,251**	**0.97 (0.52-1.53)**	**36.8%**
Heterogeneity: I^2 = 94%				
**Region = Latin America**				
Ramalho (2014), Brazil	6	43,449	1.38 (0.51-3.01)	6.5%
**Overall, Latin America**		**43,449**	**1.38 (0.51-3.01)**	**6.5%**
Heterogeneity: not applicable				
**Region = Middle East/North Africa**				
Habib (2010), Iran	28	175,235	1.60 (1.06-2.31)	8.9%
**Overall, Middle East/North Africa**		**175,235**	**1.60 (1.06-2.31)**	**8.9%**
Heterogeneity: not applicable				
**Region = North America**				
Wainer (1974), US	24	554,972	0.43 (0.28-0.64)	9.7%
Frazier (2006), US	49	944,078	0.52 (0.38-0.69)	9.9%
**Overall, North America**		**1,499,050**	**0.49 (0.38-0.61)**	**19.5%**
Heterogeneity: I^2 = 0%				
**Region = West Pacific**				
Wang (2019), China	11	418,831	0.26 (0.13-0.47)	9.6%
Su (2019), China	95	580,608	1.64 (1.32-2.00)	9.7%
Liu (1986), China	12	198,320	0.61 (0.31-1.06)	9.0%
**Overall, West Pacific**		**1,197,759**	**0.63 (0.04-1.75)**	**28.3%**
Heterogeneity: I^2 = 97%				
**Non-regionally weighted global birth prevalence**		**7,104,744**	**0.85 (0.51-1.26)**	**100.0%**
**Regionally weighted global birth prevalence[a]**			**0.96 (0.50-1.42)**	
Heterogeneity: I^2 = 96%				

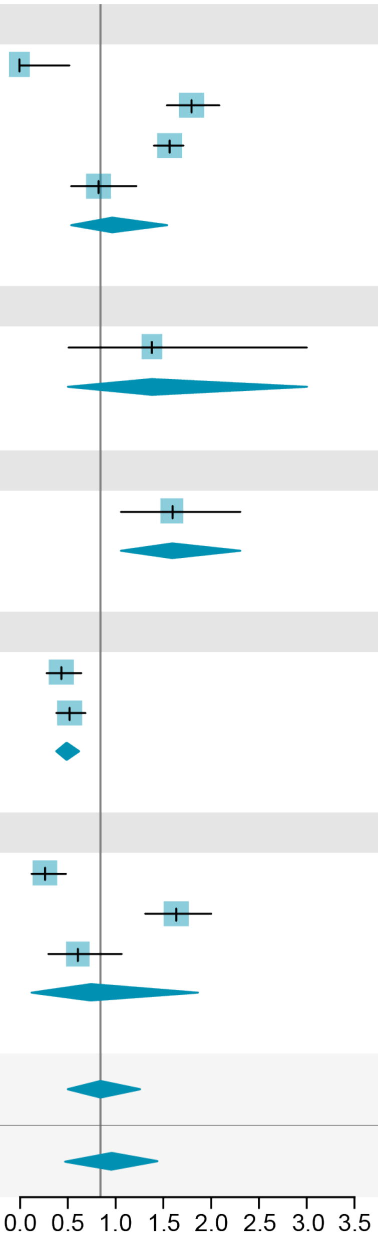

[a] The regionally weighted global prevalence was not produced directly as part of the meta-analysis but was calculated by taking a weighted average of the meta-analysis results for each of the regions, weighting them by the relative population size of each of these regions. We have added this prevalence estimate to the figure to facilitate comparison with the non-regionally weighted overall estimate from the meta-analysis.

Figure A-3. Meta-Analysis Results by Region: Confirmatory Test Phenylalanine Cutoff Value of 600 ± 100 µmol/L

Study	Cases	Screened newborns	Birth prevalence per 10,000 screened newborns (95% CI)	Weight
**Region = Europe**				
Lindner (2011), Germany	85	1,084,195	0.78 (0.63-0.97)	10.9%
Smon (2015), Slovenia	57	385,831	1.48 (1.12-1.91)	10.4%
Gerasimova (1992), USSR/Russia	26	139,664	1.86 (1.22-2.73)	9.3%
Mardesic (1986), Yugoslavia	25	274,881	0.91 (0.59-1.34)	10.2%
**Overall, Europe**		**1,884,571**	**1.18 (0.75-1.70)**	**40.8%**
Heterogeneity: I^2 = 86%				
**Region = Latin America**				
Ramalho (2014), Brazil	5	43,449	1.15 (0.37-2.69)	6.8%
Botler (2012), Brazil	26	530,248	0.49 (0.32-0.72)	10.6%
**Overall, Latin America**		**573,697**	**0.65 (0.14-1.46)**	**17.4%**
Heterogeneity: I^2 = 64%				
**Region = Middle East/North Africa**				
Abbaskhanian (2017), Iran	15	407,244	0.37 (0.21-0.61)	10.5%
**Overall, Middle East/North Africa**		**407,244**	**0.37 (0.21-0.61)**	**10.5%**
Heterogeneity: not applicable				
**Region = West Pacific**				
Boneh (2006), Australia	8	190,835	0.42 (0.18-0.83)	9.8%
Wang (2019), China	9	418,831	0.21 (0.10-0.41)	10.5%
Niu (2010), Taiwan	25	1,495,132	0.17 (0.11-0.25)	11.0%
**Overall, West Pacific**		**2,104,798**	**0.23 (0.12-0.36)**	**31.3%**
Heterogeneity: I^2 = 56%				
**Non-regionally weighted global birth prevalence**		**4,970,310**	**0.67 (0.38-1.02)**	**100.0%**
**Regionally weighted global birth prevalence[a]**			**0.50 (0.37-0.64)**	
Heterogeneity: I^2 = 94%				

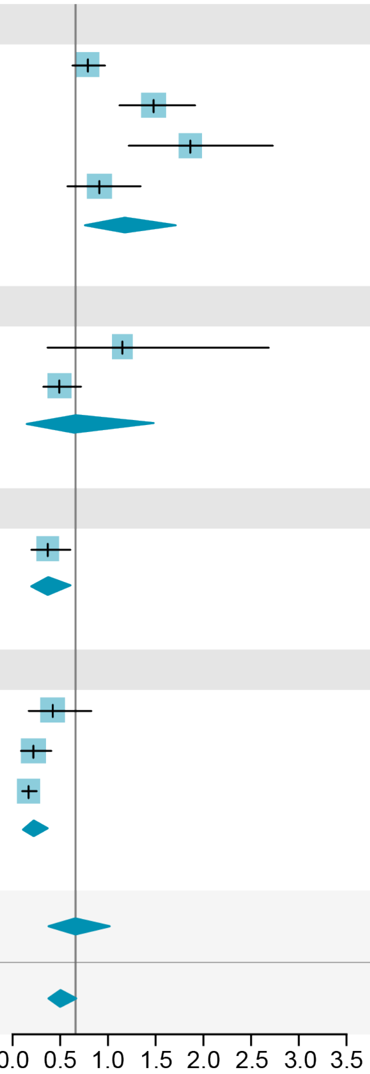

[a] The regionally weighted global prevalence was not produced directly as part of the meta-analysis but was calculated by taking a weighted average of the meta-analysis results for each of the regions, weighting them by the relative population size of each of these regions. We have added this prevalence estimate to the figure to facilitate comparison with the non-regionally weighted overall estimate from the meta-analysis.

Figure A-4. Meta-Analysis Results by Region: Confirmatory Test Phenylalanine Cutoff Value of 1,200 ± 200 μmol/L

Study	Cases	Screened newborns	Birth prevalence per 10,000 screened newborns (95% CI)	Weight
**Region = Europe**				
Ounap (1998), Estonia	6	36,074	1.66 (0.61-3.62)	3.6%
Missiou−Tsagaraki (1988), Greece	43	1,042,000	0.41 (0.30-0.56)	5.3%
Zaffanello (2002), Italy	25	1,142,338	0.22 (0.14-0.32)	5.3%
Cabalska (1993), Poland	368	2,861,504	1.29 (1.16-1.42)	5.4%
Smon (2015), Slovenia	38	385,831	0.98 (0.70-1.35)	5.2%
Gerasimova (1992), USSR/Russia	21	139,664	1.50 (0.93-2.30)	4.8%
Walker (1981), UK	39	795,382	0.49 (0.35-0.67)	5.3%
**Overall, Europe**		**6,402,793**	**0.78 (0.40-1.30)**	**34.9%**
Heterogeneity: I^2 = 97%				
**Region = Latin America**				
Ramalho (2014), Brazil	4	43,449	0.92 (0.25-2.36)	3.8%
Cornejo (2010), Chile	131	2,478,123	0.53 (0.44-0.63)	5.4%
**Overall, Latin America**		**2,521,572**	**0.58 (0.30-0.94)**	**9.2%**
Heterogeneity: I^2 = 29%				
**Region = Middle East/North Africa**				
Abbaskhanian (2017), Iran	6	407,244	0.15 (0.05-0.32)	5.2%
Karamifar (2010), Iran	3	76,966	0.39 (0.08-1.14)	4.4%
Al Hosani (2014), UAE	51	675,278	0.76 (0.56-0.99)	5.3%
**Overall, Middle East/North Africa**		**1,159,488**	**0.36 (0.04-0.94)**	**14.9%**
Heterogeneity: I^2 = 91%				
**Region = North America**				
Hansen (1978), US	39	736,469	0.53 (0.38-0.72)	5.3%
**Overall, North America**		**736,469**	**0.53 (0.38-0.72)**	**5.3%**
Heterogeneity: not applicable				
**Region = Southeast Asia**				
Pangkanon (2009), Thailand	16	5,243,841	0.03 (0.02-0.05)	5.4%
**Overall, Southeast Asia**		**5,243,841**	**0.03 (0.02-0.05)**	**5.4%**
Heterogeneity: not applicable				
**Region = West Pacific**				
Boneh (2006), Australia	1	190,835	0.05 (0.00-0.29)	5.0%
Wang (2019), China	3	418,831	0.07 (0.01-0.21)	5.2%
Su (2019), China	38	580,608	0.65 (0.46-0.90)	5.3%
Chen (1989), China	21	358,767	0.59 (0.36-0.89)	5.2%
Yoon (2005), South Korea	4	79,179	0.51 (0.14-1.29)	4.4%
Niu (2010), Taiwan	5	1,495,132	0.03 (0.01-0.08)	5.3%
**Overall, West Pacific**		**3,123,352**	**0.22 (0.03-0.56)**	**30.3%**
Heterogeneity: I^2 = 95%				
**Non-regionally weighted global birth prevalence**		**19,187,515**	**0.47 (0.26-0.74)**	**100.0%**
**Regionally weighted global birth prevalence[a]**			**0.30 (0.20-0.40)**	
Heterogeneity: I^2 = 98%				

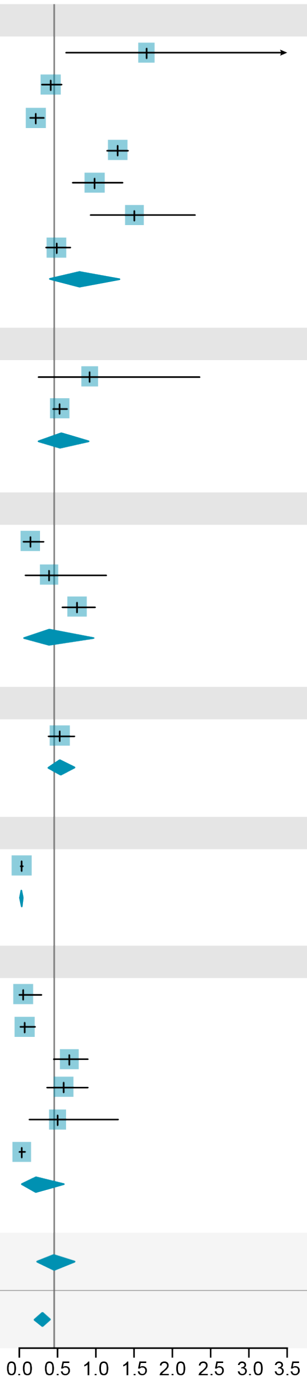

[a] The regionally weighted global prevalence was not produced directly as part of the meta-analysis but was calculated by taking a weighted average of the meta-analysis results for each of the regions, weighting them by the relative population size of each of these regions. We have added this prevalence estimate to the figure to facilitate comparison with the non-regionally weighted overall estimate from the meta-analysis.

Figure A-5. Meta-Analysis Results by Region: Overall Analysis

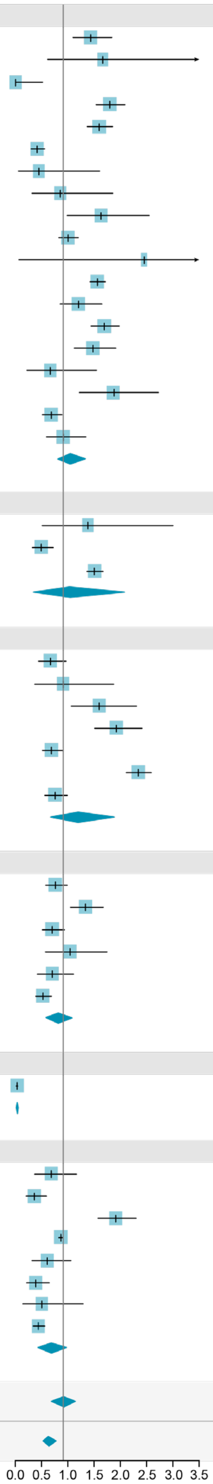

Study	Cases	Screened newborns	Birth prevalence per 10,000 screened newborns (95% CI)	Weight
**Region = Europe**				
Thalhammer (1976), Austria	61	426,141	1.43 (1.09-1.84)	2.4%
Ounap (1998), Estonia	6	36,074	1.66 (0.61-3.62)	1.6%
Visakorpi (1971), Finland	0	71,135	0.00 (0.00-0.52)	2.0%
Mathias (1986), Germany	169	940,369	1.80 (1.54-2.09)	2.5%
Lindner (2011), Germany	173	1,084,195	1.60 (1.37-1.85)	2.5%
Missiou−Tsagaraki (1988), Greece	43	1,042,000	0.41 (0.30-0.56)	2.5%
Loukas (2010), Greece	2	45,000	0.44 (0.05-1.61)	1.8%
Mehes (1985), Hungary	6	70,328	0.85 (0.31-1.86)	2.0%
Antonozzi (1980), Italy	19	116,301	1.63 (0.98-2.55)	2.2%
Zaffanello (2002), Italy	114	1,142,338	1.00 (0.82-1.20)	2.5%
Kocova (2016), Macedonia	1	4,072	2.46 (0.06-13.68)	0.4%
Cabalska (1993), Poland	447	2,861,504	1.56 (1.42-1.71)	2.5%
Vilarinho (2010), Portugal	38	316,243	1.20 (0.85-1.65)	2.4%
Dluholucky (2013), Slovakia	157	927,524	1.69 (1.44-1.98)	2.5%
Smon (2015), Slovenia	57	385,831	1.48 (1.12-1.91)	2.4%
Fernandez−Iglesias (1995), Spain	5	75,488	0.66 (0.22-1.55)	2.0%
Gerasimova (1992), USSR/Russia	26	139,664	1.86 (1.22-2.73)	2.2%
Walker (1981), UK	54	795,382	0.68 (0.51-0.89)	2.5%
Mardesic (1986), Yugoslavia	25	274,881	0.91 (0.59-1.34)	2.4%
**Overall, Europe**		**10,754,470**	**1.14 (0.89-1.41)**	**41.1%**
Heterogeneity: I^2 = 92%				
**Region = Latin America**				
Ramalho (2014), Brazil	6	43,449	1.38 (0.51-3.01)	1.7%
Botler (2012), Brazil	26	530,248	0.49 (0.32-0.72)	2.4%
Cornejo (2010), Chile	374	2,478,123	1.51 (1.36-1.67)	2.5%
**Overall, Latin America**		**3,051,820**	**0.98 (0.29-2.03)**	**6.7%**
Heterogeneity: I^2 = 96%				
**Region = Middle East/North Africa**				
Abbaskhanian (2017), Iran	27	407,244	0.66 (0.44-0.96)	2.4%
Karamifar (2010), Iran	7	76,966	0.91 (0.37-1.87)	2.0%
Habib (2010), Iran	28	175,235	1.60 (1.06-2.31)	2.3%
Motamedi (2017), Iran	74	384,933	1.92 (1.51-2.41)	2.4%
Alfadhel (2017), Saudi Arabia	53	775,000	0.68 (0.51-0.89)	2.5%
Ozalp (2001), Turkey	376	1,605,582	2.34 (2.11-2.59)	2.5%
Al Hosani (2014), UAE	51	675,278	0.76 (0.56-0.99)	2.5%
**Overall, Middle East/North Africa**		**4,100,238**	**1.18 (0.64-1.87)**	**16.5%**
Heterogeneity: I^2 = 97%				
**Region = North America**				
Hansen (1978), US	56	736,469	0.76 (0.57-0.99)	2.5%
Wainer (1974), US	74	554,972	1.33 (1.05-1.67)	2.4%
Kelly (1967), US	46	659,267	0.70 (0.51-0.93)	2.5%
Maccready (1964), US	14	134,580	1.04 (0.57-1.75)	2.2%
Zytkovicz (2001), US	18	257,000	0.70 (0.42-1.11)	2.3%
Frazier (2006), US	49	944,078	0.52 (0.38-0.69)	2.5%
**Overall, North America**		**3,286,366**	**0.81 (0.58-1.07)**	**14.4%**
Heterogeneity: I^2 = 82%				
**Region = Southeast Asia**				
Pangkanon (2009), Thailand	16	5,243,841	0.03 (0.02-0.05)	2.5%
**Overall, Southeast Asia**		**5,243,841**	**0.03 (0.02-0.05)**	**2.5%**
Heterogeneity: not applicable				
**Region = West Pacific**				
Boneh (2006), Australia	13	190,835	0.68 (0.36-1.16)	2.3%
Wang (2019), China	15	418,831	0.36 (0.20-0.59)	2.4%
Su (2019), China	111	580,608	1.91 (1.57-2.30)	2.4%
Zhan (2009), China	1,638	18,956,221	0.86 (0.82-0.91)	2.5%
Liu (1986), China	12	198,320	0.61 (0.31-1.06)	2.3%
Lin (2019), China	14	364,545	0.38 (0.21-0.64)	2.4%
Yoon (2005), South Korea	4	79,179	0.51 (0.14-1.29)	2.0%
Niu (2010), Taiwan	65	1,495,132	0.43 (0.34-0.55)	2.5%
**Overall, West Pacific**		**22,283,671**	**0.68 (0.43-0.98)**	**18.9%**
Heterogeneity: I^2 = 94%				
**Non-regionally weighted global birth prevalence**		**48,720,406**	**0.96 (0.75-1.19)**	**100.0%**
**Regionally weighted global birth prevalence[a]**			**0.64 (0.53-0.75)**	
Heterogeneity: I^2 = 98%				

[a] The regionally weighted global prevalence was not produced directly as part of the meta-analysis but was calculated by taking a weighted average of the meta-analysis results for each of the regions, weighting them by the relative population size of each of these regions. We have added this prevalence estimate to the figure to facilitate comparison with the non-regionally weighted overall estimate from the meta-analysis.
